# RACIAL DIFFERENCES IN THE RELATIONSHIP BETWEEN LONELINESS AND COGNITION AMONG OLDER ADULTS IN THE MIDWEST

**DOI:** 10.1093/geroni/igad104.3549

**Published:** 2023-12-21

**Authors:** Wade Catt, Nicole Fowler, Brea Perry, Siyun Peng, Monica Williams-Farrelly

**Affiliations:** Indiana University School of Medicine, Indianapolis, Indiana, United States; Indiana University School of Medicine, Indianapolis, Indiana, United States; Indiana University, Bloomington, Indiana, United States; Indiana University, Bloomington, Indiana, United States; Indiana University School of Medicine, Indianapolis, Indiana, United States

## Abstract

Findings from various studies have revealed a relationship between loneliness and negative health outcomes, including cognitive decline and dementia. The strength and direction of this relationship has been contested as there is wide variability in definitions and testing criteria for loneliness. If loneliness is risk factor for cognitive decline, it may represent a cost-effective site for interventional design. We used data from the Precision Health Initiative’s Person to Person Health Interview Study (P2P), a cross-sectional survey conducted in Indiana from 2020-2021, to investigate the relationship between loneliness (UCLA 3-item loneliness scale) and cognition (The Montreal Cognitive Assessment; MoCA) among older adults and to determine if the strength of the relationship varies for black and white adults. Among our subsample of adults 55 and older, over one quarter (26.7%) reported loneliness, with white respondents reporting more loneliness than black respondents (26.8 and 22.0%, respectively). Being lonely was associated with lower cognition, as was being older, male, black, and having no college education. However, we found that loneliness was associated with worse cognition, for white adults only. Although black respondents in our sample reported more loneliness than older white adults after age 70, we did not have adequate power to determine if advanced age moderated the relationship. Our findings highlight the role of loneliness in cognition for older, white adults and the need for more research to assess this relationship for the “mid-“ and “oldest-old” black adults who may be more susceptible to loneliness due, in part, to racial disparities in mortality.

